# Multi-Material Additive Manufacturing of High Temperature Polyetherimide (PEI)–Based Polymer Systems for Lightweight Aerospace Applications

**DOI:** 10.3390/polym15030561

**Published:** 2023-01-21

**Authors:** Ved S. Vakharia, Hunter Leonard, Mrityunjay Singh, Michael C. Halbig

**Affiliations:** 1 NASA Pathway Intern, Department of Mechanical and Aerospace Engineering, University of California San Diego, La Jolla, CA 92092, USA; 2 Beta Technologies, Burlington, VT 05403, USA; 3 Ohio Aerospace Institute, Cleveland, OH 44142, USA; 4 NASA Glenn Research Center, Cleveland, OH 44135, USA

**Keywords:** fused-deposition modeling, mechanical properties, polyetherimide (PEI), multi-material

## Abstract

Rapid innovations in 3-D printing technology have created a demand for multifunctional composites. Advanced polymers like amorphous thermoplastic polyetherimide (PEI) can create robust, lightweight, and efficient structures while providing high-temperature stability. This work manufactured ULTEM, a PEI-based polymer, and carbon-fiber-infused ULTEM multi-material composites with varying layering patterns (e.g., AAABBB vs. ABABAB) using fused filament fabrication (FFF). The microstructure of fractured surfaces and polished cross-sections determined that the print quality of layers printed closer to the heated bed was higher than layers closer to the top surface, primarily due to the thermal insulating properties of the material itself. Mechanical properties of the multi-material parts were between those of the single-material parts: an ultimate tensile strength and elastic modulus of 59 MPa and 3.005 GPa, respectively. Multi-material parts from the same filaments but with different layering patterns showed different mechanical responses. Prints were of higher quality and demonstrated a higher elastic modulus (3.080 GPa) when consecutive layers were printed from the same filament (AAABBB) versus parts with printed layers of alternating filaments (ABABAB), which showed a higher ultimate strength (62.04 MPa). These results demonstrate the potential for creatively designing multi-material printed parts that may enhance mechanical properties.

## 1. Introduction

The accessibility and affordability of sizeable 3-D printing systems have opened the field of materials and manufacturing to many new possibilities. Polymer matrix composites with metal additives are being used to introduce multi-functionality to parts with complex geometries. Carbon additives are being used to enhance the durability of 3-D printed parts without adding significant weight. These, among others, are examples of 3-D printed materials being investigated for their potential to provide manufacturing materials and methods for aerospace applications. The future of aviation is limited by the weight of an aircraft’s many complicated components, and additive manufacturing aims to address that challenge.

Fused filament fabrication (FFF), alternately known as fused deposition modeling (FDM), is an additive manufacturing (AM) process described as “a material extrusion process used to make thermoplastic parts through heated extrusion and deposition of materials layer by layer” [1]. FFF is one of the most widely used 3-D printing methods and has been the center of much additive manufacturing research. FFF has made great progress recently, particularly due to multi-material printing. In recent efforts, several studies have explored using FFF to 3-D print parts that integrate multiple materials [2,3,4,5,6,7,8,9,10,11,12]. Multi-material printing presents a huge potential for new multi-functionality and enhancement of overall mechanical properties of designs that may have been weak as a single-material part. Higher strength or functional materials can be directly deposited where they are needed based on the design.

Multi-material extrusion does, however, present limitations and challenges. The possibility of weak bond strength between layers of different materials presents a significant challenge that needs to be addressed. The bonding in a multi-material part can be influenced by a variety of variables, such as the many different printing parameters (heat, print speed, and layer thickness) or the properties of the materials themselves (thermal expansion rate and melting temperature) [13].

Traditionally, most of the multi-material AM research is limited to a few materials: acrylonitrile butadiene styrene (ABS), polylactic acid (PLA), and high-impact polystyrene (HIPS). These three materials are easily accessible, biodegradable, partially biocompatible, and have high impact resistance, heat resistance, and toughness [14,15,16,17]. There is significant research into reinforcing these polymers with metal and non-metal additions such as copper-infused PLA or ABS with carbon nanotubes [18,19,20,21,22].

A specific direction aviation research is currently taking is that of an electric aircraft with lighter, cooler, and more efficient motors and energy storage systems. While motors currently can reach the 95% efficiency range, motors and batteries have struggled with a heat management problem and a weight problem due to the required heavy safety systems. Potential mitigation of these problems can be addressed by replacing metals with lightweight yet strong polymers that can be 3-D printed to allow for new shapes and designs that help move heat away from sources by active or passive systems. New shapes from additive manufacturing can allow water cooling or forced air cooling of motor stators and battery cores, helping to keep systems lighter and running more efficiently at higher altitudes. PLA, ABS, and HIPS should be further investigated, but other polymers can prove more useful.

A thermoplastic material that offers the potential for applications in many engineering fields is ULTEM 9085, due to its high strength, low weight, flame retardancy, chemical resistance, and low toxicity. ULTEM 9085 can be acquired as a filament from various vendors for use in FFF. In this filament form, it exhibits a glass transition temperature (T_g_) of 186 °C [23]. However, there has been limited research conducted on this material and its application as a 3-D printed material. Some studies have investigated the effects that FFF processing parameters may have on the mechanical performance of 3-D printed ULTEM 9085 parts [24,25,26]. ULTEM 9085 has even been used to successfully print perforated engine access doors and acoustic panels and liners [27]. However, no literature exists on the use of ULTEM 9085 in a multi-material 3-D printed part. There is potential to make ULTEM 9085 parts tougher or multi-functional with the inclusion of a second material.

In this study, single- and multi-material parts were 3-D printed from ULTEM 9085 and carbon-fiber-infused ULTEM 9085. The microstructure, tensile strength, and elastic modulus were determined as a function of the FFF layering patterns. In the multi-material parts, different layering patterns were used (i.e., AAABBB vs. ABABAB) to study any effects the patterns may have on the mechanical properties. This research aimed to determine the effectiveness of using FFF to print multi-material ULTEM 9085 parts through careful observation of microstructure evolution and mechanical testing. Polymers like ULTEM 9085 show the potential to create strong, lightweight, and efficient structures to advance innovations in aviation.

## 2. Materials and Methods

A HyRel Hydra 645 (HYREL 3D, Norcross, GA, USA) was used in this work to print ASTM D638 [28] tensile specimens. The materials used, in the form of continuous filaments, were ULTEM AM9085F (SABIC, Riyadh, Saudi Arabia) and ULTEM 9085 CF (3DXTech, Grand Rapids, MI, USA) and will hereby be referred to as ULTEM 9085 and ULTEM 9085CF. The continuous filaments have a diameter of 1.75 mm and come from the supplier wound on a spool. ULTEM 9085CF is an ULTEM 9085-matrix composite with chopped carbon-fiber additions. Single-material and multi-material parts were made. The filaments used in the multi-material parts were ULTEM 9085 + ULTEM 9085CF. Each multi-material part was printed multiple times, with ULTEM 9085 + ULTEM 9085CF having different layering patterns each time. For example, one part was printed with an AAABBB pattern while another with an ABABAB pattern. The printed multi-material parts and their layering patterns are shown in Table A1.

Thermogravimetric analysis (TGA) was performed with a TGA Q500 thermogravimetric analyzer (TA Instruments, New Castle, DE, USA) for bulk filament pieces to estimate the wt.% addition of reinforcement and observe differences in impurities present in the filaments. The instrument was calibrated for temperature, heat flow, and weight according to the manufacturer’s suggestions. Specimens were cut from the filament spool and tested. TGA scans were run under air from room temperature (25 °C) to 600 °C at the heating rate of 10 °C/min. The sample purge was 60 cc/min, and the balance purge was 40 cc/min under the flowing nitrogen gas of 20 psi. Samples in the weight range of 3–5 mg were placed in a high-temperature platinum pan and degraded as a function of temperature; their mass loss was recorded by the TGA Universal Software (TA Instruments, New Castle, DE, USA).

**Figure 1:**
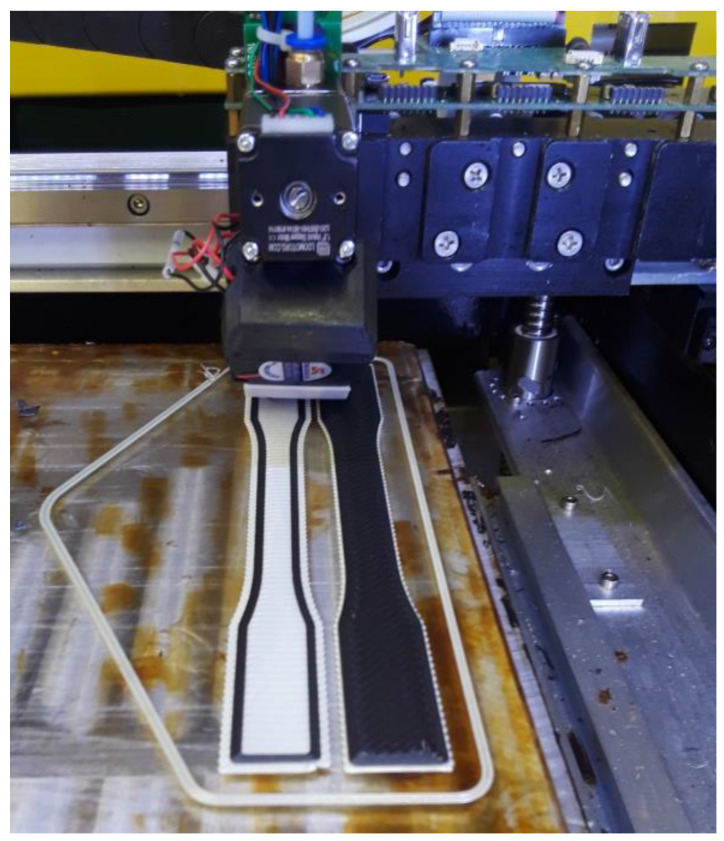
Hyrel Hydra with a MK1-450 head printing ULTEM 9085CF on an ULTEM 9085 raft on a 200 °C glass bed with a solution of PVA glazed on top.

All parts were printed at 390 °C with an MK1-450 (HyRel) head. ULTEM 9085 and ULTEM 9085CF filaments were printed with a 1.0 mm nozzle. Parts were printed with three outer perimeters, 100% infill, and a layer height half the nozzle’s width. Perimeters are used to create a well-defined part exterior to ensure the part is made accurate to the specific dimensions of the design, and the rest of the part is printed within this outline. The bed was preheated to 200 °C for ULTEM 9085 materials. A coating of PVA was applied to the print bed to increase bed adhesion. Tensile samples were 3 mm thick with six 0.5 mm layers. Parts were printed at alternating raster angles of 0° and 45°. The use of more print layers was investigated, but the final parts were printed with only 6 layers at 0.5 mm for two reasons: 3 mm provides a typical coupon thickness for mechanical testing, and the print quality diminishes with increased number of layers because the part is built up further from the heated bed. The final shapes of the parts were made according to the ASTM standards for tensile testing. Figure 1 shows the printing of an ULTEM 9085CF part.

Samples cut from printed coupons of ULTEM 9085, ULTEM 9085CF, and ULTEM 9085 + ULTEM 9085CF were mounted in epoxy and polished for microstructural analysis of the cross-sections with an optical microscope. Measurements of % area porosity of the polished cross-sections were conducted on the optical micrographs using ImageJ (National Institutes of Health, Bethesda, MD, USA). A Keyence VR-3200 profilometer (Keyence, Itasca, IL, USA) was used to develop 3-D scans of the fracture surfaces of the tensile specimens.

**Figure 2:**
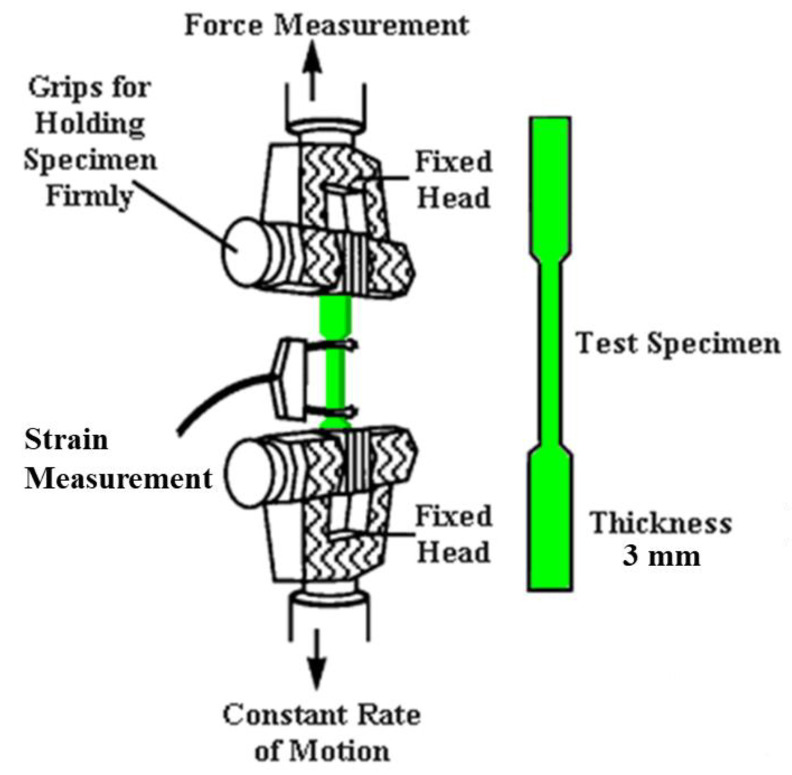
Visualization of tensile testing set up of specimens made in this study.

**Figure 3:**
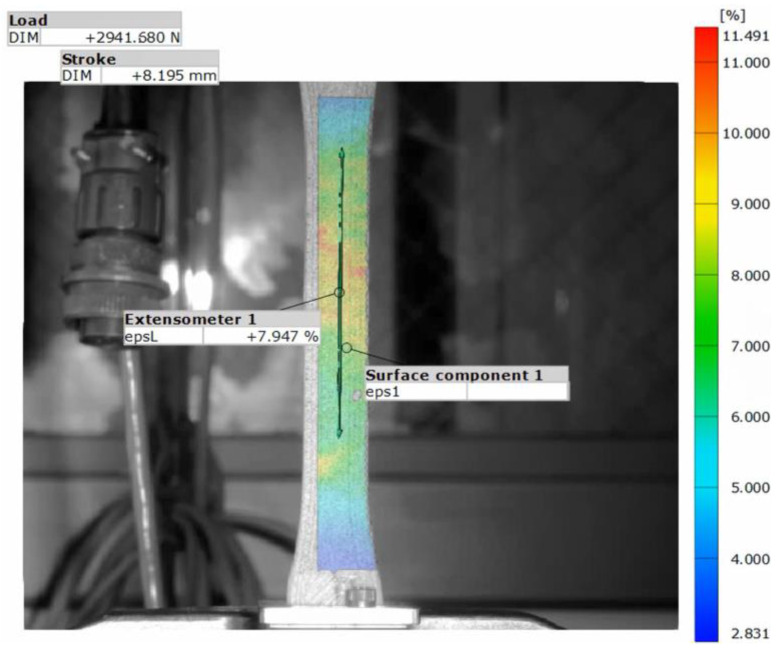
Example of DIC analysis of tensile test specimen.

For tensile testing, the strains were determined with an ARAMIS (Trilion Quality Systems, Plymouth Meeting, PA, USA) photogrammetry system using Digital Image Correlation (DIC). DIC is a non-contact optical method used to measure deformation and strain in the specimens during the tensile tests. A visualization of the test setup and an example of the DIC analysis is shown in Figure 2 and Figure 3, respectively. Mechanical modulus and ultimate tensile strength (UTS) values were determined from the tensile data. Six coupons of pure ULTEM 9085, three of ULTEM 9085CF, and two of each layering pattern (ABBABB, ABABAB, AAABBB) underwent tensile testing. Details of the material system, print layer height, coupon dimensions, elastic modulus, and ultimate strength for each tested coupon are displayed in Table A1.

## 3. Results and Discussion

### 3.1. Thermal Stability

**Figure 4:**
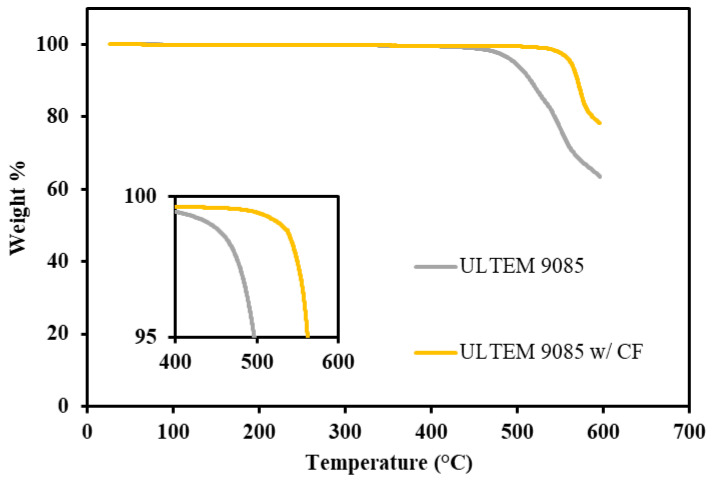
TGA results conducted in air with first 5% of weight loss magnified (inset).

The thermal stability of the filaments used in this study was investigated to characterize the high-temperature capabilities of the printed parts. Figure 4 shows the TGA (thermogravimetric analysis) results performed in air. The initial degradation temperatures (wt.% loss = 5%) for ULTEM 9085 and ULTEM 9085CF are 496 °C and 562 °C, respectively. The initial degradation temperature of ULTEM 9085 is comparable to other work, done by Padovano et al. and Kafi et al., that has investigated ULTEM 9085 parts additively manufactured with the FFF process [29,30]. Though the TGA runs in this study did not surpass 600 °C, ULTEM 9085 is expected to reach full degradation at approximately 700 °C. Compared to common FFF materials such as ABS and PLA, ULTEM materials begin degradation at higher temperatures as expected because they are known to be high-temperature polymers [22,31].

ULTEM 9085 and ULTEM 9085CF exhibit a two-step thermal degradation which the two points of inflection can note at 570 °C and 580 °C, respectively. The two-step degradation can be explained by the compositional makeup of ULTEM 9085 materials, which is a blend of PEI and polycarbonate. According to a previous study by Feng et al., polycarbonate exhibits a single-step degradation completed at approximately 510 °C [32]. The first degradation step in ULTEM 9085 materials can, therefore, be credited to the degradation of the polycarbonate present in the material. The remaining polymer in the matrix is PEI, which will achieve complete degradation at approximately 700 °C.

ULTEM 9085CF maintains a higher remaining wt% by the end of the TGA test at 600 °C, specifically, there is 78 wt% remaining compared to 64 wt% for ULTEM 9085. The matrix of the ULTEM 9085CF materials should be functionally the same as the ULTEM 9085 material, as ULTEM 9085 is a material trademarked by SABIC. Therefore, the matrix of each material should behave the same during a TGA run. The higher 12 wt% present in the ULTEM 9085CF material can be attributed to the chopped carbon fibers added during the processing of the filament. ULTEM 9085CF, compared to ULTEM 9085, is not necessarily more stable at higher temperatures.

### 3.2. Microstructral Characterization

**Figure 5:**
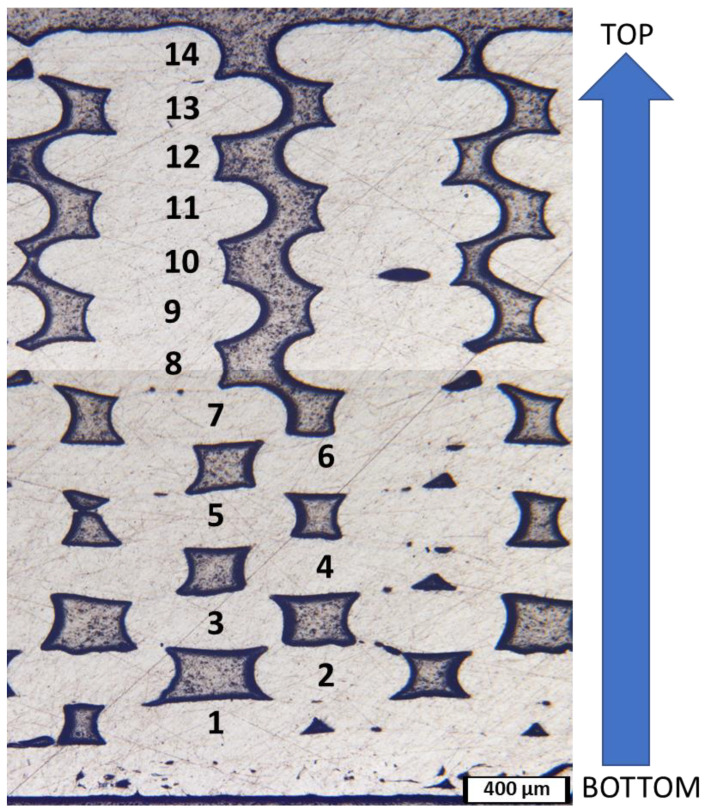
Micrograph of ULTEM 9085 polished cross-section showing all printed layers. Layers are labeled 1 through 14 with 1 being the bottom layer.

A micrograph of an entire through-thickness cross-section of an ULTEM 9085 part is shown in Figure 5. The print direction is perpendicular to the cross-section face (going into the image). As each line of the filament is printed, there is a separation of about 200 µm to account for the material expansion upon being heated during deposition. This part was intentionally printed with 14 layers to investigate the quality of each print layer in reference to its distance from the heated bed. Only the first layer exhibits enough lateral thermal expansion to account for the spacing between each pass. The second layer shows some lateral expansion, but every layer above the second shows little to no expansion. The ninth layer and above has such a slight expansion that it does not fully adhere to a second location on the previous layer and forms an empty crevice in the part that extends from the top of the eighth layer to the top surface. The poor print quality in the upper half of this part and to minimize effects of heat loss are two of the reasons why tensile specimens were made with only six print layers.

**Figure 6:**
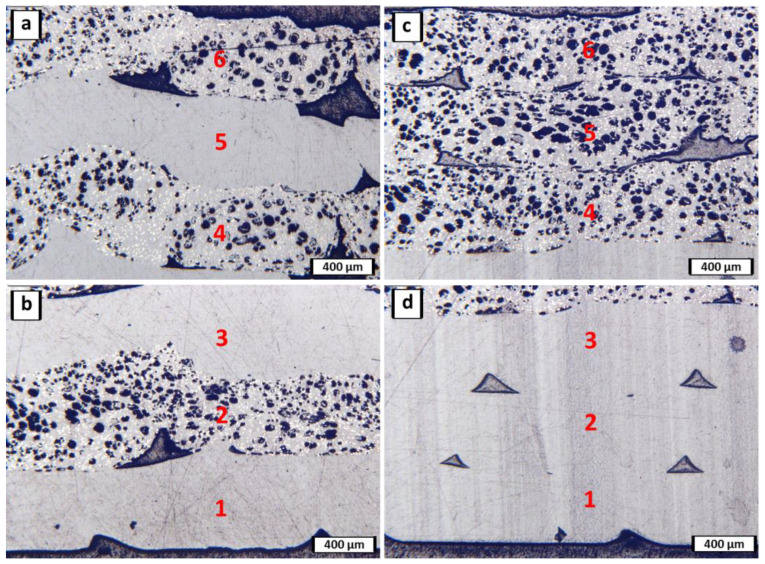
Micrographs of the top and bottom layers of ULTEM 9085 + ULTEM 9085 CF multi-material with layering pattern ABABAB (**a**,**b**) and AAABBB (**c**,**d**). Layers are labeled 1 through 6 with 1 being the bottom layer.

Micrographs of the cross-section of the multi-material ULTEM 9085 + ULTEM 9085CF parts are shown in Figure 6, with layering patterns ABABAB (Figure 6a,b) and AAABBB (Figure 6c,d). Part ABABAB exhibits inferior adhesion between layers of different materials, which in this case is every layer interface. Interestingly, the matrix of both materials is ULTEM 9085, but they are sourced from different vendors. However, it is likely that the porosity produced by the presence of chopped carbon fiber is partially causing poor adhesion between layers. The black spots in the image are pores, and the tiny white speckles are carbon fibers. Previous work has shown that the incorporation of chopped carbon fibers into a polymer-matrix led to a more porous filament which contributed to the poor adhesion between layers of a 3-D printed part, specifically in materials sourced from 3DXTech [31].

Using ImageJ, a similar porosity analysis was done, and the area % porosities of Figure 6a–d were found to be approximately 13%, 18%, 31%, and 2%, respectively. If the porosity is measured for the part’s entire cross-section, then the area % porosities of ABABAB and AAABBB are approximately 16% and 29%, respectively. Interestingly, the layers of pure ULTEM 9085 in AAABBB exhibit less porosity than those in the ABABAB part. While samples printed to 14 layers, as shown in Figure 5, may experience a significant heat loss in the upper half of the part, the parts shown in Figure 6 are only six layers thick and are still well heated through the thickness. The porosity is not an effect of heat loss in the individual printed layers. However, the materials’ heat transfer and conduction properties differ and can prevent proper adhesion or flow in the alternating layers. Another reason for the printed filament’s poor adhesion could be the material’s insufficient flow. The wetting behavior of the chopped carbon fibers in ULTEM 9085CF, and the fibers themselves, may impede proper flow of the polymer matrix if there is significant interlocking of fibers. In Part AAABBB, there is strong adhesion between the ULTEM 9085 layers. Consecutive layers are well bonded such that a layer interface cannot be identified except by the location of the few empty spaces. These patterned pores are developed between print lines of the same layer that are not printed close enough to each other and allow a gap. In Part AAABBB, the ULTEM 9085CF layers are not well-adhered to each other.

In all parts (Figure 5 and Figure 6), the adhesion between layers worsens with each subsequent layer added. For example, the adhesion between layers 1 and 2 is much better than between layers 5 and 6. The material acts as an insulator between the print bed and the topmost layer. Thus, there is a lack of heat at the higher layers, preventing proper printing behavior of the high-temperature polymer. The print chamber was not at a sufficiently high enough temperature to prevent rapid cooling of the printed layers. Without proper heating capabilities, the surface of the top layer is not hot enough to adhere well to the subsequently printed layer. ULTEM 9085 is a material that must be printed at a high temperature to ensure proper adhesion. The high print quality of the consecutive ULTEM 9085 layers in Figure 6d show that the printing parameters are properly chosen for this polymer matrix, and the poor adhesion of ULTEM 9085CF layers are more attributed to its inherent porosity, differing heat transfer coefficients, and the wetting behavior of the chopped carbon fibers. The printed parts in this study are the best obtainable parts within the confides of the printer’s capabilities. Porosity within parts containing ULTEM 9085CF is considered a design constraint, and not a function of improper printing techniques.

**Figure 7:**
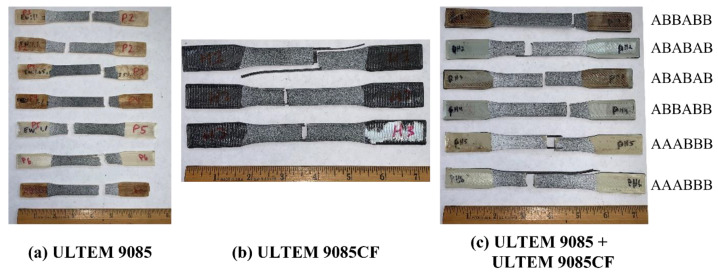
(**a**) ULTEM 9085, (**b**) ULTEM 9085CF, and (**c**) ULTEM 9085 + ULTEM 9085CF specimens after undergoing fracture during tensile testing.

**Figure 8:**
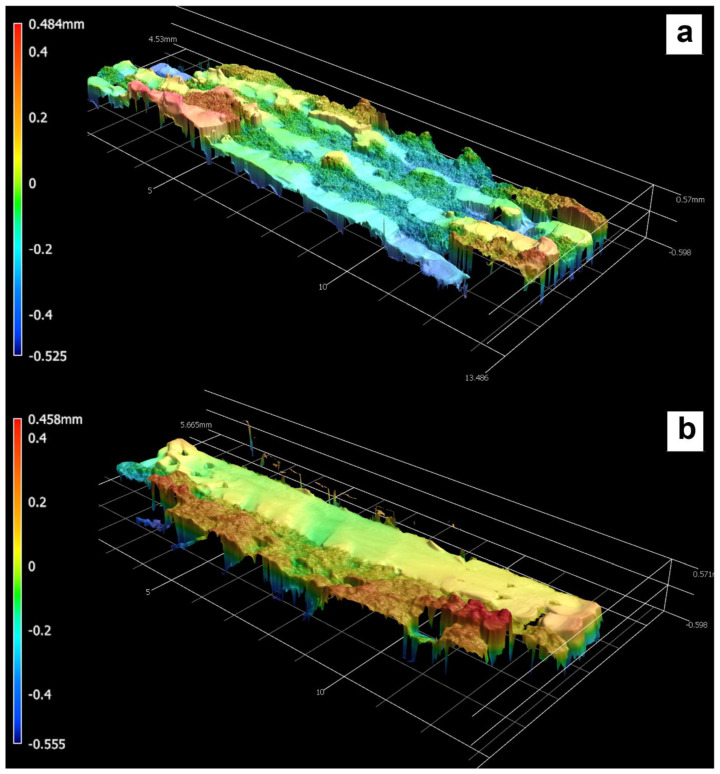
3-D scans of fracture surfaces of multi-material ULTEM 9085 + ULTEM 9085 CF parts with layering patterns ABABAB (**a**) and AAABBB (**b**).

Some of the single and multi-material specimens after fracture are displayed in Figure 7. From a macroscopic view, the individual fractures look very similar, and differing details cannot be readily determined. However, a closer look at the fracture surfaces illuminates some key differences. 3-D scans of the fracture surfaces of the multi-material ULTEM 9085 + ULTEM 9085CF parts are shown in Figure 8. Figure 8a,b show the fracture surfaces of the ABABAB and AAABBB parts, respectively. In the multi-material parts, layers of ULTEM 9085 show a relatively flat fracture surface compared to the ULTEM 9085CF layers. In ULTEM 9085 layers, we see a mostly uniform color, showing that there is not much height change on the layer surface. The opposite is seen in the ULTEM 9085CF layers; the wide range of colors implies an uneven surface. The ULTEM 9085CF layers are jagged, regardless of the layering pattern. The difference in surface roughness can be explained by the significant porosity and the brittleness of the ULTEM 9085 CF layers. The porosity acts to deflect crack propagation but also increases the number of micro-cracks that are associated with the main crack [33]. A ductile material will deform in the presence of a crack and prevent spontaneous crack growth. In contrast, a brittle material cannot deform as much and allows spontaneous crack growth [34].

**Figure 9:**
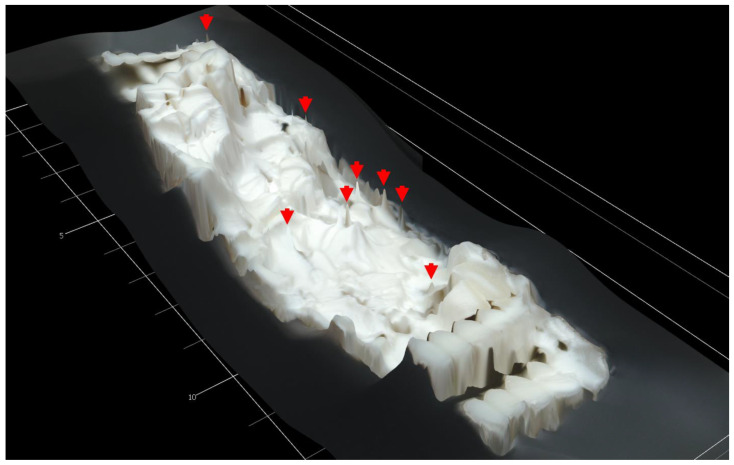
3-D scan of fracture surface of single-material ULTEM 9085 part. Conical shapes formed by necking during plastic strain shown by red arrows.

The rough fracture surface is also expected of brittle materials, whereas materials with a lower elastic modulus exhibit necking before fracture. Very ductile fractures usually form in a cone shape associated with the necking of a material, as is evident in the ULTEM 9085 part in Figure 9. The tougher ULTEM 9085 material allows for more plastic strain before failure. However, the brittle and stiff ULTEM 9085 CF layers in the multi-material parts prevented necking. The resulting fracture surfaces show smooth ULTEM 9085 layers and jagged ULTEM 9085CF layers.

### 3.3. Mechanical Properties

**Figure 10:**
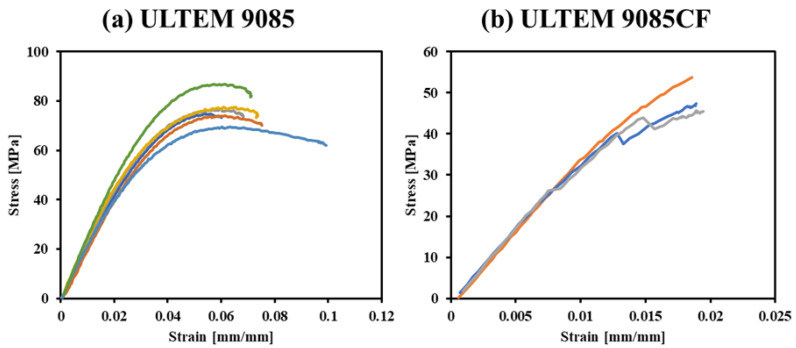
Tensile testing data for all single-material (**a**) ULTEM 9085 and (**b**) ULTEM 9085CF specimens.

Stress-strain curves of the single material specimens are shown in Figure 10. The overall results for ULTEM 9085 parts (Figure 10a) follow expected trends for plastic materials, are reasonably consistent, and provide confidence in the reproducibility of the printed parts. Like most other polymeric materials, the strain-to-failure is slightly variable. One specimen showed significantly more necking than the others, and another achieved an abnormally high max stress. From looking at the individual dimensions and mass of each specimen, accessible in Table A1, the specimens that exhibited the large necking region and higher max stress had a significantly higher mass than the rest. The heavier specimens were 10.1 g compared to 9.7 g, the average of the other four. This difference in mass may explain what allowed such plastic strain or high max loading to occur before failure. However, the two abnormal specimens behave oppositely to each other while having similar masses. It is difficult to ascertain what caused the prolonged necking or loading, and it is possible that the specimens were incorrectly placed in the testing setup. The outliers will significantly affect the average max loading and ultimate tensile strength.

Two specimens of ULTEM 9085CF (Figure 10b) underwent a discontinuity in the tensile response, which is common in some ductile materials. The material reaches an upper yield stress limit, undergoes strain before reaching a lower yield stress limit, and then experiences plastic strain. However, one of the three specimens shows a trend shared with brittle materials where the material undergoes little to no plastic strain before failure. The upper/lower yield stress limits and the little to no plastic strain before failure are characteristics of ductile and brittle materials, respectively, so ULTEM 9085CF can be characterized as a material type somewhere between brittle and ductile. From the micrographs of ULTEM 9085CF in Figure 6, it is clear there is significant porosity present in the material. Porosity often introduces variation in a material’s mechanical behavior, which may be the reason for the variation in stress–strain trends exhibited by ULTEM 9085CF.

**Figure 11:**
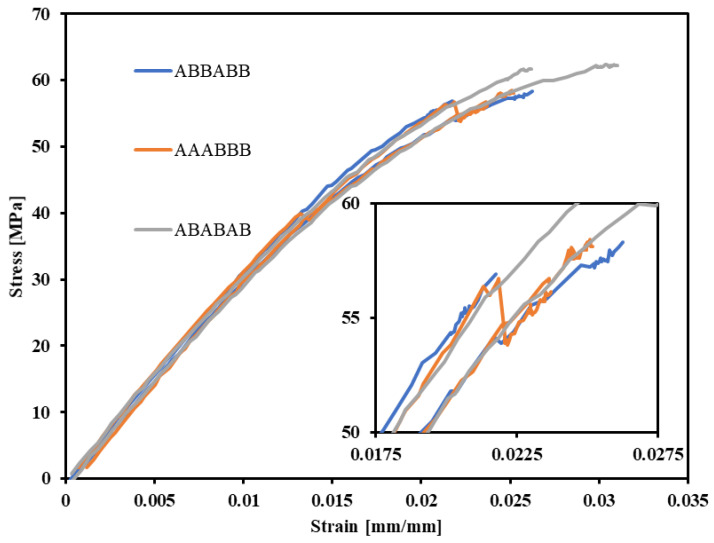
Tensile testing data for all multi-material A—ULTEM 9085 + B—ULTEM 9085CF specimens. Magnification of strain near fracture is shown in the inset.

Stress–strain curves of the multi-material specimens are shown in Figure 11. Multiple runs of the same coupon type are displayed to show the slight variability between tests. Though there is some inconsistency between individual runs, they generally follow the same trend. The multi-material of AAABBB layering exhibits an upper and lower yield stress limit, similar to the ULTEM 9085CF parts. However, it also plastically strains past that yield stress, similar to the ULTEM 9085 parts. The remaining multi-material parts show brittle characteristics; there is no necking before failure and little plastic strain. Regardless of the layering pattern, all multi-material parts exhibit similar elastic moduli.

**Figure 12:**
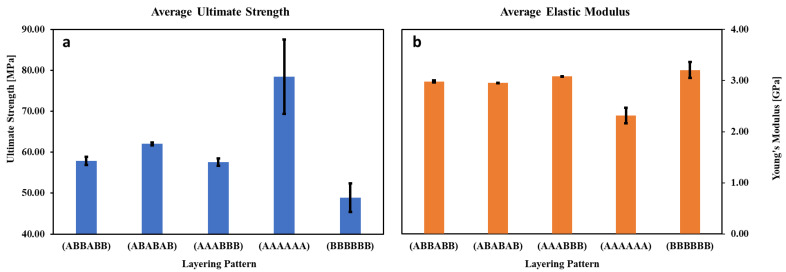
Average (**a**) ultimate tensile strengths and (**b**) elastic moduli of single and multi-material parts made from A—ULTEM 9085 + B—ULTEM 9085CF.

The average elastic moduli and ultimate strengths of the singular-material ULTEM 9085 and the ULTEM 9085 + ULTEM 9085CF multi-material parts are shown in Figure 12 and mechanical properties of the specimens can be found in Table A1; the layering patterns differentiate the multi-material parts. The standard deviation represents how widely the values range from the mean. Specifically, it was calculated with the following formula:

(1) $$\sqrt{\frac{\sum^{\;}{\left(x-\bar{x}\right)}^{2}}{n}},$$

where $\bar{x}$ is the average value and n is the sample size. The large standard deviation in the mechanical properties of the pure ULTEM 9085 coupon (AAAAAA) is caused by an outlier coupon that exhibited an ultimate strength and elastic moduli of 98 MPa and 2.584 GPa, respectively. If that coupon is discounted, the average ultimate strength and elastic moduli of the pure ULTEM 9085 parts would be 75 MPa (±2.8 MPa) and 2.262 GPa (±0.103 GPa), respectively. However, the large deviation in the pure ULTEM 9085CF coupons (BBBBBB) should not be disregarded. Porosity in the as-received ULTEM 9085CF filament presents unpredictability and variability in the end print quality of the parts. The broader range of potential strength and modulus values is an important design aspect to consider when designing structural parts with this material.

The ultimate strengths and elastic moduli observed in the ULTEM 9085 parts of this study are within the range of strengths observed in multiple studies [28,35,36,37]. As expected, the ULTEM 9085CF part is the stiffest (3.204 GPa), primarily due to the high modulus of chopped carbon fiber, while the ULTEM 9085 part is the most ductile. Similarly, ULTEM 9085 has the higher ultimate strength (78 MPa) and toughness, and ULTEM 9085CF has the lower (49 MPa). The low ultimate tensile strength of ULTEM 9085CF parts is partially due to the large porosity within the layers. There is potential for ULTEM 9085CF parts to be significantly more robust if the porosity can be minimized. ULTEM 9085 parts exhibited significantly more plastic strain before failure than the ULTEM 9085CF parts. All multi-material parts are in-between the two extremes in strength and stiffness (elastic modulus). The ULTEM 9085CF layers have a more significant influence on the stiffness of the multi-material part than the ULTEM 9085 layers. The multi-material parts exhibit an elastic modulus less than 300 MPa lower than the ULTEM 9085CF part but approximately 700 MPa higher than the ULTEM 9085 parts. In the case of ultimate strength, the multi-material parts exhibit strengths much closer in value to the ULTEM9085CF single-material specimens.

Interestingly, ABABAB patterned parts exhibit a lower elastic modulus but a higher ultimate strength than AAABBB patterned parts. AAABBB parts have three consecutive layers of a plastic material (ULTEM 9085) followed by three of a ductile-brittle material (ULTEM 9085CF). The three consecutive, porous ductile-brittle layers could allow crack propagation before necking begins with less resistance than the plastic ULTEM 9085 layers, which may cause the earlier failure. In comparison, the ABABAB parts show more plastic strain because the stiffer carbon-fiber-infused layers are interrupted by plastic ULTEM 9085 layers and prevent early tensile failure.

**Figure 13:**
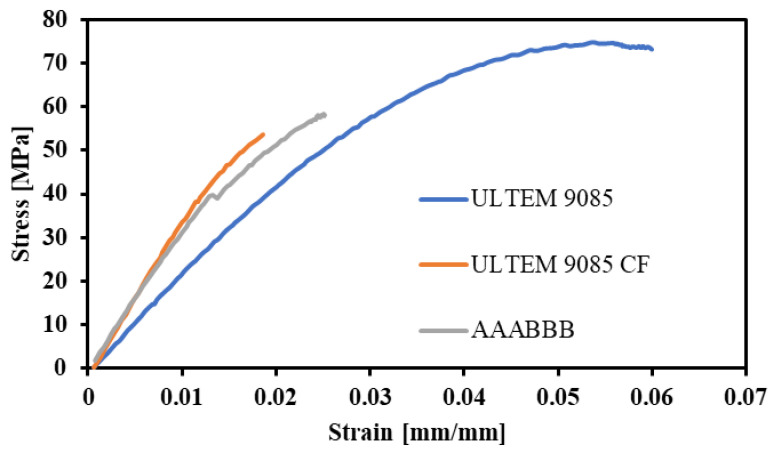
Stress–strain data of the single-material and multi-material (AAABBB) parts made from ULTEM 9085 and ULTEM 9085 CF.

In order to compare the three material types, Figure 13 shows one stress–strain curve example of each type of specimen. ULTEM 9085, being a more plastic material, exhibits necking and a vast plastic strain region, whereas ULTEM 9085CF does not. The chopped carbon fiber and the porosity within the ULTEM 9085 matrix create a more brittle material with a higher elastic modulus. Similarly, the multi-material part does not exhibit much necking or a plastic strain region, which corroborates the conclusions made from the 3-D scans of fracture surfaces in Figure 8. The brittle carbon fiber layers of the multi-material are strong enough to prevent the necking usually exhibited by the ULTEM 9085 layers. The differences in elastic moduli and ultimate strengths are explained by a combination of the sequential order of the brittle and ductile layers and the significant presence of porosity.

## 4. Conclusions

In this study, multi-material parts were printed with ULTEM 9085 and ULTEM 9085CF. Upon studying the microstructure and the interface between consecutive layers, there is a clear difference in print quality between the first and last layers printed. ULTEM 9085 is appealing for its high thermal resistance. However, that same property is why heat does not efficiently conduct from the print bed to the highest print layers, resulting in poor application and adhesion of the material. A high-temperature material, such as ULTEM 9085, is problematic to easily 3-D print with the FFF process, and introducing a second material only complicates the optimization process.

Furthermore, ULTEM 9085CF has a large amount of apparent porosity: approximately 31% area compared to 2% area for pure ULTEM 9085 layers. This extensive porosity results in poor adhesion, independent of the consecutive layers’ likeness or difference in materials, whereas successive layers of ULTEM 9085 adhere reasonably well until the printed layers are approximately 1.5 mm from the print bed. Print parameters can affect the quality of prints, but in this study, the print settings are reasonably chosen, as is shown by the high print quality of the pure ULTEM 9085 layers. However, the print chamber should be maintained at a high enough temperature to not allow significant cooling of the part during the printing process.

However, even with the print difficulties, it is evident that the order in which a multi-material part is printed, AAABBB vs. ABABAB, has a pronounced effect on the resulting mechanical properties. This work has provided insight into how carbon-fiber-infused layers prevent necking in printed ULTEM 9085 parts while also inducing a higher ultimate tensile strength. The toughness of the different materials plays a role in the fracture behavior of the tensile coupons. While a ULTEM 9085 part would experience prolonged plastic strain or necking at the fracture surface due to its high plasticity, the introduction of the brittle carbon-fiber-infused ULTEM 9085 layers prevents this necking. Instead, it exhibits behavior more consistent with a brittle fracture. The mechanical response is explained by the individual mechanical characteristics of the two different ULTEM 9085 materials. The mechanical behavior can be predicted with a strong understanding of how the different brittle and ductile materials behave under tensile stress. Multi-material parts with alternating ULTEM 9085 and ULTEM 9085 CF layers exhibit more strain before failure than those with consecutive ULTEM 9085 layers (0.033 vs. 0.025 mm/mm), most likely due to the toughness of the ULTEM 9085 layers allowing continued elongation. Less plastic strain is observed in AAABBB parts, presumably due to the brittle ULTEM 9085 CF layers causing an early brittle fracture in half of the specimen or the micro-cracks formed from the large porosity.

Further studies into multi-material printing, particularly with a high-temperature polymer such as polyetherimide, may add to the vast potential of additive manufacturing in the aeronautical and space industries. This study shows the potential for introducing multifunctionality, enhancing the already superb mechanical or thermal properties, and optimizing the printing parameters of promising part designs.

## Data Availability

Not applicable.

## Appendix A

**Table A1:** Details of all specimens used in this study.

Material	Layering	Width	Depth	Mass	Max Load	UTS	Elastic Modulus
		mm	mm	g	N	MPa	GPa
ULTEM 9085	AAAAAA	12.3	3.3	9.6	3012	75	2.250
ULTEM 9085	AAAAAA	12.6	3.4	9.7	3164	74	2.178
ULTEM 9085	AAAAAA	12.5	3.0	9.8	2903	77	2.360
ULTEM 9085	AAAAAA	12.7	2.9	9.6	2817	77	2.395
ULTEM 9085	AAAAAA	12.5	3.2	10.1	2974	70	2.125
ULTEM 9085	AAAAAA	12.5	2.8	10.1	3387	98	2.584
ULTEM 9085CF	BBBBBB	12.9	2.9	8.0	1795	47	3.227
ULTEM 9085CF	BBBBBB	12.8	2.9	7.9	1992	54	3.381
ULTEM 9085CF	BBBBBB	12.8	2.9	8.2	1688	46	3.005
ULTM 9085 + ULTEM 9085CF	ABBABB	13.1	3.5	9.7	2577	57	3.005
ULTM 9085 + ULTEM 9085CF	ABBABB	12.9	3.5	9.7	2631	59	2.960
ULTM 9085 + ULTEM 9085CF	ABABAB	13.0	3.3	9.8	2653	62	2.950
ULTM 9085 + ULTEM 9085CF	ABABAB	12.9	3.4	9.8	2679	62	2.956
ULTM 9085 + ULTEM 9085CF	AAABBB	13.0	3.0	8.9	2180	57	3.083
ULTM 9085 + ULTEM 9085CF	AAABBB	13.0	2.9	8.8	2164	58	3.077
